# Glutaric acid associates with early detection of cognitive impairment deciphering the association with depression

**DOI:** 10.1002/alz.088825

**Published:** 2025-01-09

**Authors:** Manas Chakraborty, Kunal Dhiman, Perminder S. Sachdev, Henry Brodaty, Brian Draper, Julian N Trollor, Simone Reppermund, Kristan Kang, Karen A Mather, Anne Poljak, Wei Wen, Katya T. Numbers, Nicole A. Kochan, Vibeke S. Catts, Veer Gupta

**Affiliations:** ^1^ Deakin University, Waurn Ponds, VIC Australia; ^2^ Deakin University, Highton, VIC Australia; ^3^ Centre for Healthy Brain Ageing (CHeBA), University of New South Wales, UNSW Sydney, NSW Australia; ^4^ Centre for Healthy Brain Ageing (CHeBA), UNSW Sydney, Sydney, NSW Australia; ^5^ Centre for Healthy Brain Ageing, Discipline of Psychiatry and Mental Health, University of New South Wales, Sydney, NSW Australia; ^6^ Centre for Healthy Brain Ageing, University of New South Wales, Sydney Australia; ^7^ Centre for Healthy Brain Ageing (CheBA), School of Psychiatry, Kensington, NSW Australia; ^8^ Deakin University, Geelong, VIC Australia

## Abstract

**Background:**

The initiation of cognitive impairment is triggered by a myriad of pathological events occurring decades before clinical symptoms manifest. Perturbed glucose and fatty acid metabolism notably contribute to the development of cognitive impairment, progressing further into clinical dementia. These metabolic alterations are evident in plasma through changes in specific metabolites. Notably, these changes are characteristic features in the pathophysiology of depression, a significant risk factor for cognitive impairment. This project aims to establish a blood‐based biomarker signature for early identification of cognitive changes in individuals with depression. Moreover, it aims to assist in diagnosing and understanding disease progression by quantifying plasma metabolite levels linked to fatty acid metabolism in samples obtained from participants in the Sydney Memory and Ageing Study.

**Method:**

Plasma samples underwent analysis to quantify fatty acids and carnitines using chemical derivatization through UPLC‐MRM/MS coupled with a 4000 QTRAP mass spectrometer which was equipped with an electrospray ion (ESI) source and operated in the multiple‐reaction monitoring (MRM) mode with negative‐ion (‐) detection.10‐μL aliquots of each of the resultant calibration solutions and each of the sample solutions were injected for analysis.

**Result:**

ANCOVA, revealed significant changes in the levels of several biomarkers including glycolic acid (p‐value: 0.033), C181 carnitine (p‐value: 0.013), glutaric acid (p‐value: 0.08) levels in groups with Mild Cognitive Impairment (MCI) and MCI with comorbid depression compared to healthy groups. ROC curve analysis demonstrated that glutaric acid effectively distinguished between MCI and non‐MCI participants. The optimal threshold for classifying participants into MCI and non‐MCI groups was determined using the Youden Index to dichotomize them based on high and low biomarker levels. Logistic regression analysis, adjusting for age, sex, APOE ε4 genotype, and comorbid depression, revealed a significant association between glutaric acid levels and cognitive impairment in the context of depression, with a p‐value of <0.001 and an odds ratio of 2.49.

**Conclusion:**

Glutaric acid proved to be a reliable discriminator between participants with MCI and those without MCI. The aforementioned findings revealed a strong correlation between glutaric acid and cognitive impairment associated with depression which could be used as biomarker for early detection of cognitice impairment.

